# Hospital-Diagnosed Infections, Autoimmune Diseases, and Subsequent Dementia Incidence

**DOI:** 10.1001/jamanetworkopen.2023.32635

**Published:** 2023-09-07

**Authors:** Janet Janbek, Thomas Munk Laursen, Niels Frimodt-Møller, Melinda Magyari, Jürgen G. Haas, Richard Lathe, Gunhild Waldemar

**Affiliations:** 1 Danish Dementia Research Centre, Department of Neurology, Copenhagen University Hospital–Rigshospitalet, Copenhagen, Denmark; 2 National Centre for Register-Based Research, Department of Economics and Business Economics, Aarhus BSS, Aarhus University, Aarhus, Denmark; 3 Department of Clinical Microbiology, Copenhagen University Hospital– Rigshospitalet, Copenhagen, Denmark; 4 Department of Clinical Medicine, University of Copenhagen, Copenhagen, Denmark; 5 Danish Multiple Sclerosis Center, Department of Neurology, Copenhagen University Hospital–Rigshospitalet, Glostrup, Denmark; 6 Division of Infection Medicine, University of Edinburgh, Edinburgh, United Kingdom

## Abstract

**Importance:**

Systemic inflammation has been suggested to explain reported associations between infections and dementia. Associations between autoimmune diseases and dementia also suggest a role for peripheral systemic inflammation.

**Objective:**

To investigate the associations of infections and autoimmune diseases with subsequent dementia incidence and to explore potential shared signals presented by the immune system in the 2 conditions.

**Design, Setting, and Participants:**

This nationwide, population-based, registry-based cohort study was conducted between 1978 and 2018 (40-year study period). All Danish residents born 1928 to 1953, alive and in Denmark on January 1, 1978, and at age 65 years were included. Persons with prior registered dementia and those with HIV infections were excluded. Data were analyzed between May 2022 and January 2023.

**Exposures:**

Hospital-diagnosed infections and autoimmune diseases.

**Main Outcomes and Measures:**

All-cause dementia, defined as the date of a first registered dementia diagnosis after age 65 years in the registries. Poisson regression with person-years at risk as an offset variable was used to analyze time to first dementia diagnosis.

**Results:**

A total of 1 493 896 individuals (763 987 women [51%]) were followed for 14 093 303 person-years (677 147 [45%] with infections, 127 721 [9%] with autoimmune diseases, and 75 543 [5%] with dementia). Among individuals with infections, 343 504 (51%) were men, whereas among those with autoimmune diseases, 77 466 (61%) were women. The dementia incidence rate ratio (IRR) following any infection was 1.49 (95% CI, 1.47-1.52) and increased along with increasing numbers of infections in a dose-dependent manner. Dementia rates were increased for all infection sites in the short term, but not always in the long term. The dementia IRR following any autoimmune disease was 1.04 (95% CI, 1.01-1.06), but no dose-dependent increase was observed, and only a few autoimmune conditions showed increased IRRs for dementia.

**Conclusions and Relevance:**

These findings may point toward a role for infection-specific processes in the development of dementia, rather than general systemic inflammation, as previously hypothesized. Assessing these 2 conditions in a single setting may allow for additional insights into their roles in dementia and for hypotheses on possible underlying mechanisms.

## Key Points

**Question:**

Is exposure to infections and/or autoimmune diseases associated with dementia incidence?

**Findings:**

In this nationwide cohort study of 1 493 896 individuals, infections were associated with a statistically significant 1.49-fold increased dementia incidence in a dose-dependent manner, particularly in the short term. Associations were less clear for autoimmune diseases.

**Meaning:**

The observed associations of infections with dementia and the lack of such associations for autoimmune disease may point toward a role for infection-specific processes, rather than general systemic inflammation, as recently hypothesized.

## Teaser

This cohort study investigates the associations of infections and autoimmune diseases with subsequent dementia incidence and explores potential shared signals presented by the immune system in the 2 conditions.

## Introduction

There has been extensive debate about whether infections might be causally linked to dementia, particularly Alzheimer disease (AD).^1^ Evidence from epidemiological and other studies has several limitations. First, studies have generally focused on specific infections and/or pathogens; therefore, it is unclear whether specific infectious agents might be involved or whether inflammation induced by infection could underlie the reported observed risks. Second, most studies investigated associations in postmortem brains and were, hence, challenged by temporality. Third, most studies had short follow-up periods and were based on selected populations. Four recent population-based studies^2,3,4,5^ showed an increased risk of dementia following different types of infection (hospital and/or primary care diagnoses). Two reported an increased risk of dementia and/or AD following any infection^3,5^ and suggested that systemic inflammation, rather than specific infections or pathogens, might explain the observed increase in risk.

A large body of literature supports a role for systemic inflammation in increased dementia risk and includes epidemiological studies on diseases that induce a proinflammatory state.^6^ Epidemiological studies assessing links between autoimmune diseases and dementia share a common hypothesis that peripheral systemic inflammation may increase the risk of dementia. Most studies investigated individual autoimmune diseases (or groups) and found mixed results.^7^ The evidence, therefore, remains inconclusive.

By investigating infections and autoimmune diseases together, we aimed to explore potential shared signals presented by the immune system in these different but mainly inflammatory conditions that could advance knowledge on their roles as dementia risk factors and on the possible underlying mechanisms. The aim was, therefore, to investigate the association between infections and autoimmune diseases and dementia incidence.

## Methods

### Data Sources

We used population-based Danish national registries. The National Patient Register (NPR) and the Psychiatric Central Research Register hold records from somatic (from 1977) and psychiatric (from 1969) wards, respectively, with outpatient data added in 1995. The National Prescription Register contains all prescription drugs dispensed at Danish pharmacies from 1995.^8^ No diagnostic codes from primary care are directly recorded in the health registers. We defined our variables using either hospital diagnostic codes or prescriptions or their combination. Danish law does not require ethical approval or informed consent for registry-based studies. The study followed Strengthening the Reporting of Observational Studies in Epidemiology (STROBE) reporting guidelines for cohort studies.

### Study Design and Population

This population-based cohort study was conducted from 1978 to 2018. Included were all individuals born from 1928 to 1953 who were alive and in Denmark on January 1, 1978, and on the date of their 65th birthday. Persons with prior recorded dementia and those with HIV infections were excluded.

### Exposure: Infections and Autoimmune Diseases

Exposure was defined as inpatient, outpatient, or emergency hospital contacts with a primary or secondary discharge diagnosis of an infection or autoimmune disease (codes are shown in eTable 1 and eTable 2 in Supplement 1) in the NPR from age 50 years onward (based on inpatient data only before 1995). Infections were assessed as follows: any (ie, presence of the date of a first registered diagnosis; reference, no infection); burden (detailed in the eAppendix in Supplement 1), defined as the number of new infections and of inpatient admissions (reference, no infection); time since first infection within 5 years and greater than 5 years (reference, no infection) to test for associations in the short and long term, respectively; and infection site (ie, date of a first registered diagnosis of an infection of each site as defined in eTable 1 in Supplement 1; reference, no infection of the assessed site). Each person could have infections in multiple sites.

Autoimmune diseases were assessed as follows: any (ie, presence of a date of a first registered diagnosis; reference, no autoimmune disease); burden (detailed in the eAppendix in Supplement 1), defined as the number of different types of autoimmune disease and of inpatient admissions (reference, no autoimmune disease); and type of disease, defined as the presence of the date of a first registered diagnosis of an autoimmune disease of each category (eTable 2 in Supplement 1) (reference, no disease of the type assessed). Each person could have multiple diseases.

### Outcome: All-Cause Dementia

All-cause dementia was defined as the date of a first registered dementia diagnosis after age 65 years in the NPR and Psychiatric Central Research Registry (validated previously^9^), or the date of a first redeemed antidementia prescription from the National Prescription Register (codes in eTable 3 in Supplement 1). The latter was used to identify dementia cases diagnosed in the primary care setting (used as disease proxies).^10,11^

### Covariates

Covariates were sex, age, calendar year, highest attained education at age 50 years, and selected comorbidities (ie, diabetes, hypertension, stroke, myocardial infarction, and hypercholesteremia). Comorbidities were defined using hospital inpatient and outpatient diagnoses or medication prescriptions (eTable 4 in Supplement 1).

### Statistical Analysis

Poisson regression with person-years at risk as an offset variable was used to analyze time to first dementia diagnosis (approximation to Cox regression) using SAS statistical software version 9.4 (SAS Institute) and estimating incidence rate ratios (IRRs).^12,13^ Constant rates were assumed in the used periods only (age in 5-year intervals and calendar year in 1-year intervals), which were small enough periods to ensure that the constant rates were fulfilled.^12,13^ Statistical significance was determined using 2-sided *P* < .05. Individuals were followed from their 50th birthday (risk time from age 65 years). Follow-up terminated on the date of the outcome, emigration, death, or December 31, 2018, whichever came first. Exposure dates, comorbidities, age, and calendar time were analyzed time dependently (eFigure 1 in Supplement 1).

Exposures were analyzed as defined. To test the comparability of infection sites and/or autoimmune disease types with each other, we also analyzed IRRs with a common reference to no infection or no disease. Model 1 was adjusted for age, sex, calendar year, and highest attained education; model 2 was further adjusted for selected comorbidities; and the fully adjusted model was further adjusted for infections when analyzing autoimmune diseases and vice versa to test whether one was associated with the risk of the other.

Post hoc, we analyzed dementia incidence since the first infection for each infection site. Four sensitivity analyses were defined a priori to test the robustness of results. First, we analyzed IRRs in 2 calendar periods to test risk variation. Second, we estimated mortality rate ratios following exposures. Third, we removed codes that were judged to be uncertain for infection (eTable 1 in Supplement 1). Fourth, we included exposures before age 50 years. Data were analyzed between May 2022 and January 2023.

## Results

In total 1 493 896 individuals (763 987 women [51%]) were followed-up for 14 093 303 person-years (eFigure 2 in Supplement 1). During the study period, 677 147 people (45%) were registered with infections, and 127 721 (9%) were registered with autoimmune diseases, from age 50 years onward. A total of 75 543 persons (5%) were registered with all-cause dementia (from age 65 years onward; median [IQR] age at dementia incidence, 77 [72-81] years). Among individuals with infections, 343 504 (51%) were men, whereas among those with autoimmune diseases, 77 466 (61%) were women. On the date of dementia diagnosis, people with infections or autoimmune diseases were slightly older than those without (Table).

**Table.** Population Baseline Characteristics^a^

Characteristic	Participants, No. (%)				
	Total (N = 1 493 896)	Infections		Autoimmune diseases	
		Exposed (n = 677 147 [45%])	Nonexposed (n = 816 749 [55%])	Exposed (n = 127 721 [9%])	Nonexposed (n = 1 366 175 [91%])
Sex					
Female	763 987 (51)	333 643 (49)	430 344 (53)	77 466 (61)	686 521 (50)
Male	729 909 (49)	343 504 (51)	386 405 (47)	50 255 (39)	679 654 (50)
Dementia during study	75 543 (5)	36 524 (5)	39 019 (5)	6729 (5)	68 814 (5)
Age at incident dementia (≥65 y), median (IQR), y	77 (72-81)	78 (73-82)	76 (72-80)	78 (74-82)	77 (72-81)
Age at first exposure (≥50 y), median (IQR), y	NA	66 (59-72)	NA	65 (57-71)	NA

Abbreviation: NA, not applicable.

^a^ Table shows the baseline characteristics of the cohort in total, those exposed to infections, and those exposed to autoimmune diseases from age 50 years onward. Some people could have both an infection and autoimmune disease exposure but were analyzed separately in the exposure groups. In total, 82 555 people had both an infection and an autoimmune disease throughout the study period, regardless of the relative timing of the infection and autoimmune disease to each other (ie, 6% of the entire population, 65% of all those with autoimmune diseases, and 12% of all those with infections).

The median (IQR) age at first exposure was 66 (59-72) years for infection and 65 (57-71) years for autoimmune disease. Respiratory infections were the most common, followed by gastrointestinal and urinary infections. The most common autoimmune diseases were rheumatoid arthritis and polymyalgia rheumatica.

**Figure 1.**
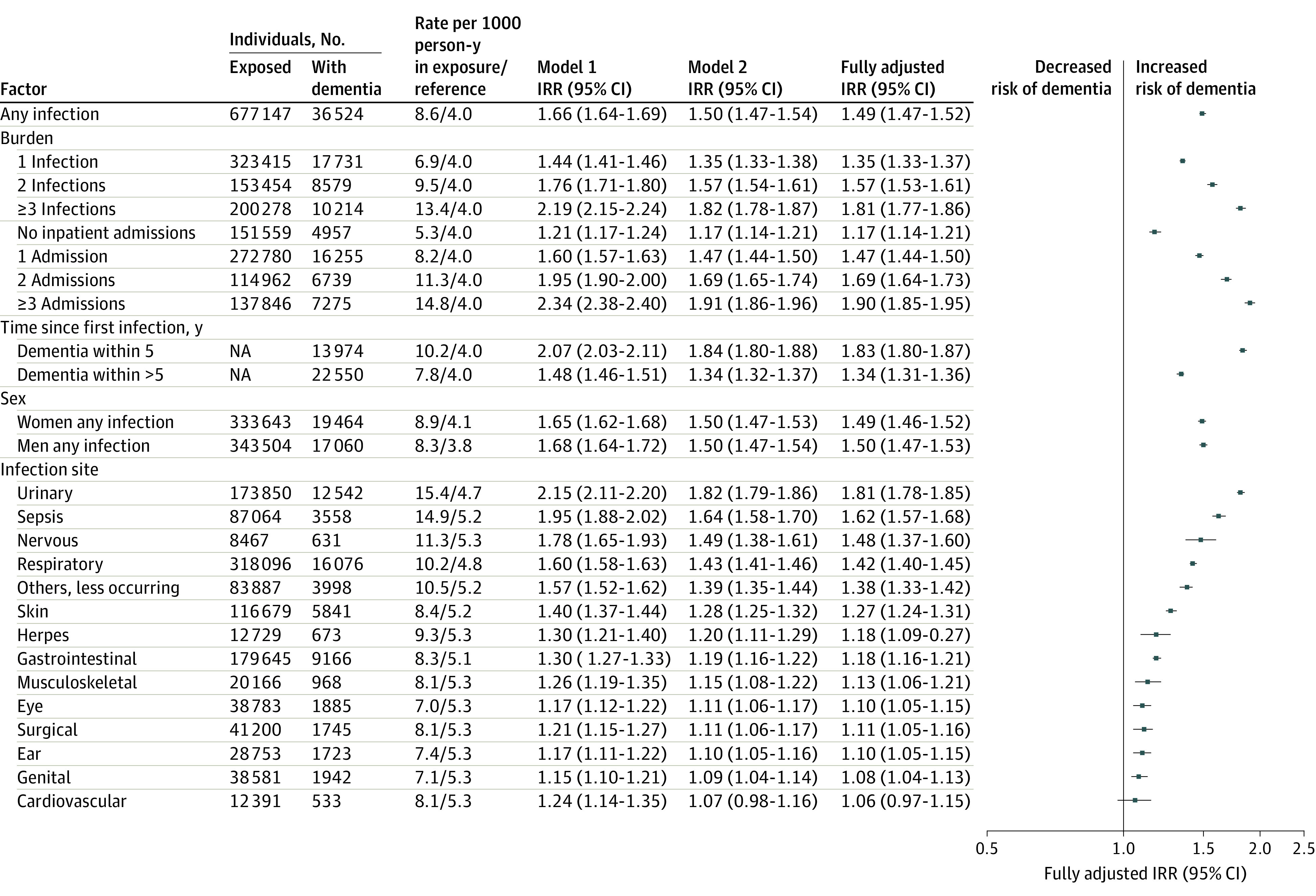
Infections and Subsequent Dementia Incidence Plot shows dementia incidence rate ratios (IRRs) for infection (reference, no infection). Model 1 IRRs were adjusted for age, sex, calendar year, and highest attained educational level. Model 2 IRRs were further adjusted for hypertension, diabetes, hypercholesteremia, myocardial infarction, and stroke. The fully adjusted IRRs were further adjusted for autoimmune diseases. The forest plot presents the fully adjusted estimates. NA indicates not applicable.

Figure 1 presents dementia IRRs following infections. The IRR was increased for persons with any infection vs those without infection (fully adjusted IRR, 1.49; 95% CI, 1.47-1.52). IRRs were similar in men and women and increased in a dose-dependent manner along with increasing burden of infection (fully adjusted IRR for ≥3 infections, 1.81; 95% CI, 1.77-1.86). The IRR was increased both within 5 years (fully adjusted IRR, 1.83; 95% CI, 1.80-1.87) and more than 5 years (fully adjusted IRR, 1.34; 95% CI, 1.31-1.36) after infection. Significantly increased IRRs were seen across all infection sites except for cardiovascular infections, with the highest for urinary infections (fully adjusted IRR, 1.81; 95% CI, 1.78-1.85).

**Figure 2.**
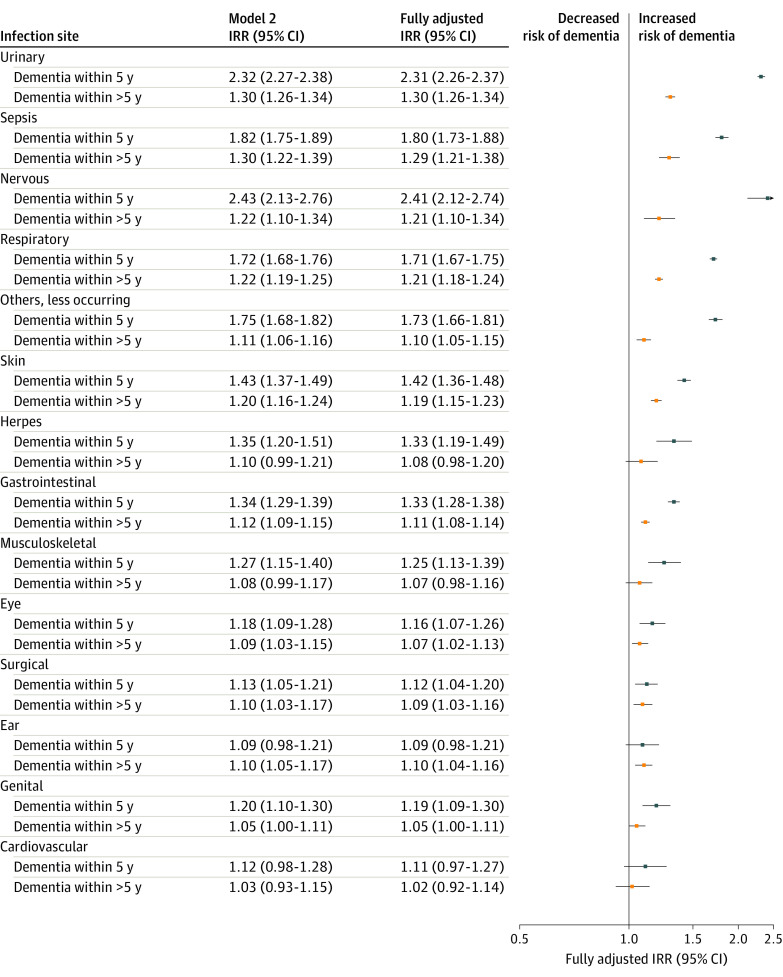
Post Hoc Analysis of Time Since First Infection of Each Site Plot shows dementia incidence rate ratios (IRRs) for each infection site when stratified by time since first infection: dementia within 5 years and greater than 5 years of first infection (reference, no infection of each site). Model 2 IRRs were adjusted for age, sex, calendar year, highest attained educational level, hypertension, diabetes, hypercholesteremia, myocardial infarction, and stroke. The fully adjusted IRRs were further adjusted for autoimmune diseases. The forest plot presents the fully adjusted estimates.

Figure 2 presents post hoc analysis of the time since the first infection at each site. Dementia IRRs were highest within 5 years and were significantly increased for all sites except for ear and cardiovascular infections. At more than 5 years after infection, IRRs were smaller but remained statistically significant across some sites.

**Figure 3.**
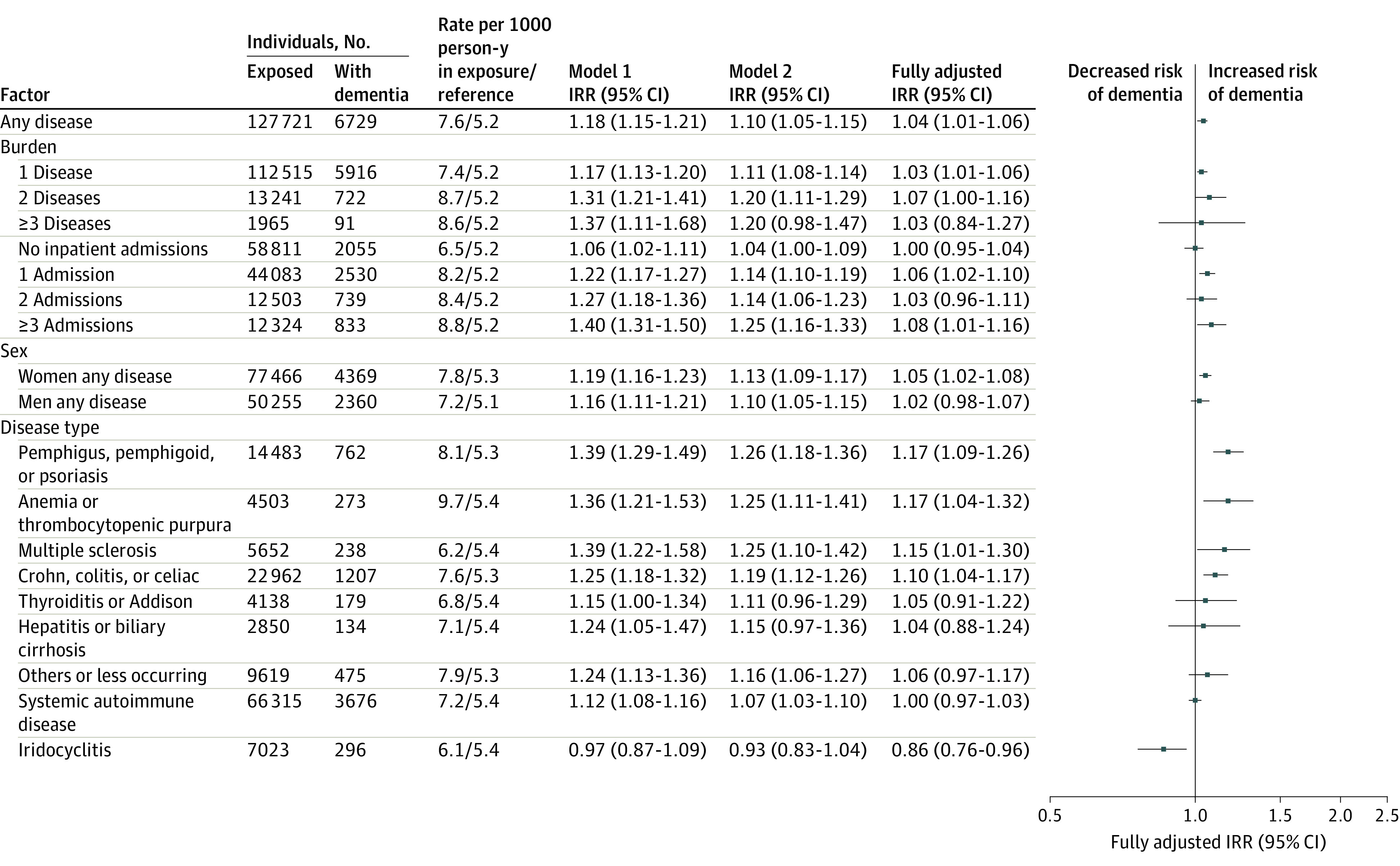
Autoimmune Diseases and Subsequent Dementia Incidence Plot shows dementia incidence rate ratios (IRRs) for any autoimmune disease (reference, no disease). Model 1 IRRs were adjusted for age, sex, calendar year, and highest attained educational level. Model 2 IRRs were further adjusted for hypertension, diabetes, hypercholesteremia, myocardial infarction, and stroke. Fully adjusted IRRs were further adjusted for infections. The forest plot presents the fully adjusted estimates. The systemic autoimmune diseases group includes seropositive rheumatoid arthritis, Wegener granulomatosis, dermatopolymyositis, polymyalgia rheumatica, systemic sclerosis, systemic lupus erythematosis, Sjogren syndrome, and ankylosing spondylitis. Anemia is autoimmune pernicious anemia.

Figure 3 presents dementia IRRs following autoimmune disease. The IRR for persons with any autoimmune disease was increased vs those without disease but was very small, especially after adjustment for infections (fully adjusted IRR, 1.04; 95% CI, 1.01-1.06). The IRR for women was slightly higher (fully adjusted IRR, 1.05; 95% CI, 1.02-1.08). No dose-response association was seen for autoimmune disease burden. Statistically significant IRRs were seen in a few disease categories, but were very small, particularly after adjustment for infections.

eTable 5 and eTable 6 in Supplement 1 show IRRs for each site or disease type when a common reference group was set. Trends were similar to those presented in Figure 1.

Sensitivity analyses showed increased mortality rate ratios following infection and autoimmune disease, but they were higher for infections than for autoimmune disease. Other analyses showed the robustness of estimates to any definitions we made (eFigure 3 in Supplement 1).

## Discussion

In this nationwide cohort study of approximately 1.5 million individuals over a period of 40 years (risk time for up to 25 years), we found that hospital-diagnosed infections were associated with increased IRRs for subsequent all-cause dementia, whereas IRRs were much smaller for autoimmune diseases. Infections were associated with a 1.49-fold increased rate of dementia. We observed a dose-response association, and dementia rates were increased in both the short and long term, although the increase was greater in the short term. Dementia rates were increased for all infection sites (except for cardiovascular) in the short term, but not always in the long term. By contrast, autoimmune diseases were associated with only a 1.04-fold increased rate of dementia, but no dose-response association was seen and only a few autoimmune disease types were associated with any increase in dementia IRR. In addition, the effect sizes were small, particularly after adjustment for infections.

### Infections

Our findings are consistent with previous population-based studies evaluating dementia risks after hospital-diagnosed infections. Sipilä et al^3^ reported a 1.5-fold increased dementia risk in Finland and a 2.6-fold increased risk in the UK biobank, Sun et al^5^ reported a 1.16-fold increased AD risk in Sweden, and Muzambi et al^2^ found a 1.99-fold increased dementia rate in the UK (all observed dose-response associations). Muzambi et al^2^ also assessed infections diagnosed in the primary care setting and found a significant but small association with dementia risk, consistent with another previous study.^4^ In all studies, as with ours, associations persisted in the long term but were much lower than in the short term, as also reported in the recent study by Levine et al.^14^

The infection sites analyzed in our study were not similarly analyzed in the previous studies. Sun et al^5^ assessed central nervous system, gastrointestinal, respiratory, genitourinary, and skin infections, whereas Muzambi et al^2^ assessed sepsis, pneumonia and other respiratory infections, and urinary and skin infections. These were also the sites that had the highest short-term and long-term IRRs in our study. Our data also showed increased rates for all other infection sites except for herpesvirus, musculoskeletal, and genital infections, which were no longer significant in the long term (in addition to already insignificant estimates for cardiovascular infections). We also observed overall smaller effect sizes in the long term across most sites, suggesting potential reverse causality. The short-term effects could indicate a role for infections in triggering, accelerating, or unmasking already existing dementia pathology and is, thus, an important clinical and public health avenue for interventions. In addition, as shown in our and the previous studies, the associations are probably not specific for central nervous system infection and do not appear to be organ, system, or pathogen specific.

Other previous studies investigated specific infections and reported increased dementia risks associated with gastrointestinal infections,^15^ sepsis,^16^ and pneumonia.^17,18^ Herpesvirus infections have gained the most attention but with mixed results, and all recent large studies except one showed no increased dementia risk.^19,20,21,22,23,24^ In our study, for any herpesvirus infection, only short-term estimates were significant.

### Autoimmune Diseases

The evidence to date from epidemiological studies is mixed. Most previous studies that investigated specific autoimmune diseases were based on small and selected populations. Population-based studies on specific autoimmune diseases (eg, inflammatory bowel diseases,^7,25,26^ psoriasis,^27^ rheumatic diseases,^28,29^ and others^7^) have reported either no or a small increase in dementia risk, especially in the long term (where assessed), although with some heterogeneity. Three previous studies investigated several types of autoimmune diseases in the UK^29,30^ and Sweden.^31^ These found increased dementia risk following only some autoimmune diseases. However, estimates generally attenuated toward the null in the long term, and any positive effect sizes were small, as in our study, and were generally higher in the Swedish study.^31^ In our study, we further noted the lack of a dose-response association, which was not investigated previously. This, together with the small IRRs, provides little evidence for an association of autoimmune disease with subsequent dementia development, especially after adjustment for infections. Autoimmune disease diagnosis may also be preceded by infections (multiple sclerosis is a recent example^32^), thus explaining the overall change in estimates after adjustment for infections. The small effect sizes reported may reflect ascertainment bias because patients with autoimmune conditions probably receive more thorough medical follow-up, which could increase the likelihood of receiving a dementia diagnosis.

### Potential Mechanisms

Previous studies^3,5^ have suggested that systemic inflammation, rather than specific pathogens, could underlie the association between infections and dementia. The likely mechanisms include a role for proinflammatory and anti-inflammatory cytokines, blood-brain barrier dysfunction,^33^ and peripheral-central immune system crosstalk.^6^ Similar mechanisms involving peripheral systemic inflammation have also been hypothesized to link some autoimmune diseases with dementia.^7,29^ Recent data from genomewide association studies^34^ suggest that autoimmunity may be associated with dementia pathology. Overall, it is increasingly suggested that immune system dysregulation leading to an overactive, underactive, and/or chronic inflammatory response may play a role in the development of dementia.

Our data and the observational nature of our study do not directly support or refute any proposed mechanism, nor can we make firm conclusions about the role of inflammation in dementia on the basis of our findings. However, the associations of infection with dementia found in our study, together with the very small IRRs for autoimmune disease, may point toward a role for infection-specific processes rather than general systemic inflammation.

Several findings suggest that a possible explanation is that a weakened immune system may predispose not only to severe infections (that require hospitalization) but also to dementia development. First, previous reports^2,4^ showed that infections that did not require hospitalization (diagnosed in the primary care setting) were not associated with increased dementia risk. Second, we have previously shown that people with dementia have higher rates of infection hospitalization,^35^ demonstrating a bidirectional association and the vulnerability to infection hospitalization. In the present study, the increased short-term but not long-term dementia risks for some infection sites lead us to theorize that the observed infection and dementia associations could be related to reverse causality. Along with the small IRRs for autoimmune disease, the evidence could possibly point away from an association between systemic inflammation and dementia development and instead could suggest that severe infections resulting from a weakened immune system, rather than inflammation per se, underlie the association with dementia. Finally, it is possible that some of the observed risks might be attributed to delirium,^36^ and we encourage future studies to assess its potential role.

### Strengths and Limitations

The major strengths of our study lie in the comprehensive investigation of both conditions, infections and autoimmune disease. To our knowledge, both conditions have been evaluated for the first time in one population and one single setting, which allows us to compare them with each other and draw important mechanistic conclusions. Further strengths of this study are its prospective and nationwide coverage, with negligible loss to follow-up or selection bias, as well as the long study period.

This study also has limitations that should be mentioned. First, we were unable to explore dementia subtypes because of diagnostic uncertainties. In studies^3,17,29,30^ that assessed subtypes, associations with autoimmune disease and/or infections were more prominent for vascular dementia than for other subtypes. These findings, if based on valid subtype definitions, are of special interest because associations between infections and dementia have principally been directed toward AD pathology, prompted by findings that Aβ peptide, the deposition of which is a hallmark of AD, has antimicrobial properties.^1^ Another major limitation was the inability to isolate associations attributed to treatment vs the conditions themselves, in part because of a lack of comprehensive treatment data. Attempts to isolate such associations should be conducted in future studies.

Importantly, we note the limitations of our data for drawing conclusions on the associations of autoimmune diseases with dementia. In most cases, autoimmune diseases are treated with long-term anti-inflammatory or immunosuppressive medications. This may mean that the small IRRs observed in our study are masked by the effect of medications either by suppressing severe inflammation (hence, inflammation per se was not assessed in our study) or reducing dementia risk, as suggested in some studies^34,37,38^ (albeit with inconclusive evidence). To acknowledge this, we assessed autoimmune disease exposure using inpatient admissions (to proxy active disease and/or inflammation). No clear dose-response association was observed because the 95% CIs overlapped between exposure groups. However, we did not have data on the severity of inflammation or disease and on treatments dispensed at hospitals. In addition, we concluded that the association of autoimmune disease with dementia is tenuous, albeit statistically significant, especially in model 1. We further point out the potential for interaction between autoimmune diseases and infections, which we did not assess.

Other limitations common to observational and registry-based studies include residual confounding from lifestyle differences and validity of diagnostic codes. We also did not have genetic data, which are likely to play an important role in the associations under investigation.^34^ Furthermore, it is not possible to conclude whether the dose-response association is present in both the short and long term, because we assessed this regardless of the timing.

## Conclusions

In this cohort study of approximately 1.5 million people over a period of 40 years, hospital-diagnosed infections, at all but 1 infection site, were associated with increased rates of dementia (particularly in the short term), and we found a dose-response association. The IRRs were much smaller for autoimmune disease. To our knowledge, this study is the first to assess all sites of infection, the long-term and short-term risks across all sites, and the burden of infection and autoimmune disease using multiple measures. Importantly, this is the first study, to our knowledge, to assess the 2 exposures in 1 nationwide cohort, allowing us to draw insights that will advance knowledge on their roles as risk factors for dementia development and on the possible underlying mechanisms.

## Notes

Supplement 1. **eAppendix.** Supplemental Methods **eTable 1.** *ICD* Codes for Infections Definition, by Infection Site **eTable 2.** *ICD* Codes for Autoimmune Diseases Definition, by Disease Type **eTable 3.** *ICD* and ATC Codes for Dementia Definition **eTable 4.** *ICD* and ATC Codes for Chosen Comorbidities **eFigure 1.** Data Analysis Illustration **eFigure 2.** Population Flow Chart **eTable 5.** Infection Sites and Subsequent Dementia (Common Reference = No Infection) **eTable 6.** Autoimmune Disease Types and Subsequent Dementia (Common Reference = No Disease) **eFigure 3.** Sensitivity Analyses
eReferences

Supplement 2.
Data Sharing Statement

## References

[1] National Institute on Aging, Division of Neuroscience. Infectious etiology of Alzheimer’s disease: is there a causative role for infectious agents in Alzheimer’s disease? October 5, 2021. Accessed August 7, 2023. https://www.nia.nih.gov/sites/default/files/2022-03/workshopsummary_infectious-etiology-ad_final.pdf

[2] Muzambi R, Bhaskaran K, Smeeth L, Brayne C, Chaturvedi N, Warren-Gash C. Assessment of common infections and incident dementia using UK primary and secondary care data: a historical cohort study. Lancet Healthy Longev. 2021;2(7):e426-e435. doi:10.1016/S2666-7568(21)00118-5 34240064 PMC8245326

[3] Sipilä PN, Heikkilä N, Lindbohm JV, . Hospital-treated infectious diseases and the risk of dementia: a large, multicohort, observational study with a replication cohort. Lancet Infect Dis. 2021;21(11):1557-1567. doi:10.1016/S1473-3099(21)00144-4 34166620 PMC8592915

[4] Douros A, Santella C, Dell’Aniello S, . Infectious disease burden and the risk of Alzheimer’s disease: a population-based study. J Alzheimers Dis. 2021;81(1):329-338. doi:10.3233/JAD-201534 33780369

[5] Sun J, Ludvigsson JF, Ingre C, . Hospital-treated infections in early- and mid-life and risk of Alzheimer’s disease, Parkinson’s disease, and amyotrophic lateral sclerosis: a nationwide nested case-control study in Sweden. PLoS Med. 2022;19(9):e1004092. doi:10.1371/journal.pmed.1004092 36107840 PMC9477309

[6] Xie J, Van Hoecke L, Vandenbroucke RE. The impact of systemic inflammation on Alzheimer’s disease pathology. Front Immunol. 2022;12:796867. doi:10.3389/fimmu.2021.796867 35069578 PMC8770958

[7] Booth MJ, Kobayashi LC, Janevic MR, Clauw D, Piette JD. No increased risk of Alzheimer’s disease among people with immune-mediated inflammatory diseases: findings from a longitudinal cohort study of U.S. older adults. BMC Rheumatol. 2021;5(1):48. doi:10.1186/s41927-021-00219-x 34763722 PMC8588609

[8] Schmidt M, Schmidt SAJ, Adelborg K, . The Danish health care system and epidemiological research: from health care contacts to database records. Clin Epidemiol. 2019;11:563-591. doi:10.2147/CLEP.S179083 31372058 PMC6634267

[9] Phung TKT, Andersen BB, Høgh P, Kessing LV, Mortensen PB, Waldemar G. Validity of dementia diagnoses in the Danish hospital registers. Dement Geriatr Cogn Disord. 2007;24(3):220-228. doi:10.1159/000107084 17690555

[10] Taudorf L, Nørgaard A, Islamoska S, Jørgensen K, Laursen TM, Waldemar G. Declining incidence of dementia: a national registry-based study over 20 years. Alzheimers Dement. 2019;15(11):1383-1391. doi:10.1016/j.jalz.2019.07.006 31587994

[11] Pottegård A, Schmidt SAJ, Wallach-Kildemoes H, Sørensen HT, Hallas J, Schmidt M. Data resource profile: the Danish National Prescription Registry. Int J Epidemiol. 2017;46(3):798. 27789670 10.1093/ije/dyw213 PMC5837522

[12] Andersen PK, Keiding N. Multi-state models for event history analysis. Stat Methods Med Res. 2002;11(2):91-115. doi:10.1191/0962280202SM276ra 12040698

[13] Laird N, Olivier D. Covariance analysis of censored survival data using log-linear analysis techniques. J Am Stat Assoc. 1981;76(374):231-240. doi:10.1080/01621459.1981.10477634

[14] Levine KS, Leonard HL, Blauwendraat C, . Virus exposure and neurodegenerative disease risk across national biobanks. Neuron. 2023;111(7):1086-1093.e2. doi:10.1016/j.neuron.2022.12.029 36669485 PMC10079561

[15] Fink A, Doblhammer G, Tamgüney G. Recurring gastrointestinal infections increase the risk of dementia. J Alzheimers Dis. 2021;84(2):797-806. doi:10.3233/JAD-210316 34602468 PMC8673498

[16] Lei S, Li X, Zhao H, . Risk of dementia or cognitive impairment in sepsis survivals: a systematic review and meta-analysis. Front Aging Neurosci. 2022;14:839472. doi:10.3389/fnagi.2022.839472 35356300 PMC8959917

[17] Chu C-S, Liang C-S, Tsai S-J, . Bacterial pneumonia and subsequent dementia risk: a nationwide cohort study. Brain Behav Immun. 2022;103:12-18. doi:10.1016/j.bbi.2022.04.002 35390468

[18] Chalitsios CV, Baskaran V, Harwood RH, Lim WS, McKeever TM. Incidence of cognitive impairment and dementia after hospitalisation for pneumonia: a UK population-based matched cohort study. ERJ Open Res. 2023;9(3):00328. doi:10.1183/23120541.00328-2022 37228284 PMC10204809

[19] Tsai M-C, Cheng W-L, Sheu J-J, . Increased risk of dementia following herpes zoster ophthalmicus. PLoS One. 2017;12(11):e0188490. doi:10.1371/journal.pone.0188490 29166672 PMC5699837

[20] Johannesdottir Schmidt SA, Veres K, Sørensen HT, Obel N, Henderson VW. Incident herpes zoster and risk of dementia: a population-based Danish cohort study. Neurology. 2022;99(7):e660-e668. doi:10.1212/WNL.0000000000200709 35676090 PMC9484607

[21] Choi HG, Park BJ, Lim JS, Sim SY, Jung YJ, Lee SW. Herpes zoster does not increase the risk of neurodegenerative dementia: a case-control study. Am J Alzheimers Dis Other Demen. Published online April 22, 2021;36:15333175211006504. doi:10.1177/15333175211006504 33882722 PMC11005322

[22] Warren-Gash C, Williamson E, Shiekh SI, . No evidence that herpes zoster is associated with increased risk of dementia diagnosis. Ann Clin Transl Neurol. 2022;9(3):363-374. doi:10.1002/acn3.51525 35170873 PMC8935278

[23] Schnier C, Janbek J, Williams L, . Antiherpetic medication and incident dementia: observational cohort studies in four countries. Eur J Neurol. 2021;28(6):1840-1848. doi:10.1111/ene.14795 33657269

[24] Murphy MJ, Fani L, Ikram MK, Ghanbari M, Ikram MA. Herpes simplex virus 1 and the risk of dementia: a population-based study. Sci Rep. 2021;11(1):8691. doi:10.1038/s41598-021-87963-9 33888766 PMC8062537

[25] Rønnow Sand J, Troelsen FS, Horváth-Puhó E, Henderson VW, Sørensen HT, Erichsen R. Risk of dementia in patients with inflammatory bowel disease: a Danish population-based study. Aliment Pharmacol Ther. 2022;56(5):831-843. doi:10.1111/apt.17119 35781292 PMC9545113

[26] Zhang MN, Shi YD, Jiang HY. The risk of dementia in patients with inflammatory bowel disease: a systematic review and meta-analysis. Int J Colorectal Dis. 2022;37(4):769-775. doi:10.1007/s00384-022-04131-9 35325272

[27] Zhao J, Li T, Wang J. Association between psoriasis and dementia: a systematic review. Neurologia (Engl Ed). Published online March 23, 2021. doi:10.1016/j.nrl.2020.12.007 38161072

[28] Lin T-M, Chen W-S, Sheu J-J, Chen Y-H, Chen J-H, Chang C-C. Autoimmune rheumatic diseases increase dementia risk in middle-aged patients: a nationwide cohort study. PLoS One. 2018;13(1):e0186475. doi:10.1371/journal.pone.0186475 29304089 PMC5755737

[29] Zhang Y-R, Yang L, Wang H-F, . Immune-mediated diseases are associated with a higher incidence of dementia: a prospective cohort study of 375,894 individuals. Alzheimers Res Ther. 2022;14(1):130. doi:10.1186/s13195-022-01072-x 36100869 PMC9472428

[30] Wotton CJ, Goldacre MJ. Associations between specific autoimmune diseases and subsequent dementia: retrospective record-linkage cohort study, UK. J Epidemiol Community Health. 2017;71(6):576-583. doi:10.1136/jech-2016-207809 28249989

[31] Li X, Sundquist J, Zöller B, Sundquist K. Dementia and Alzheimer’s disease risks in patients with autoimmune disorders. Geriatr Gerontol Int. 2018;18(9):1350-1355. doi:10.1111/ggi.13488 30044040

[32] Sedighi S, Gholizadeh O, Yasamineh S, . Comprehensive investigations relationship between viral infections and multiple sclerosis pathogenesis. Curr Microbiol. 2022;80(1):15. doi:10.1007/s00284-022-03112-z 36459252 PMC9716500

[33] Galea I. The blood-brain barrier in systemic infection and inflammation. Cell Mol Immunol. 2021;18(11):2489-2501. doi:10.1038/s41423-021-00757-x 34594000 PMC8481764

[34] Lindbohm JV, Mars N, Sipilä PN, ; FinnGen. Immune system-wide Mendelian randomization and triangulation analyses support autoimmunity as a modifiable component in dementia-causing diseases. Nat Aging. 2022;2(10):956-972. doi:10.1038/s43587-022-00293-x 37118290 PMC10154235

[35] Janbek J, Frimodt-Møller N, Laursen TM, Waldemar G. Dementia identified as a risk factor for infection-related hospital contacts in a national, population-based and longitudinal matched-cohort study. Nat Aging. 2021;1(2):226-233. doi:10.1038/s43587-020-00024-0 37118634

[36] Richardson SJ, Lawson R, Davis DHJ, . Hospitalisation without delirium is not associated with cognitive decline in a population-based sample of older people-results from a nested, longitudinal cohort study. Age Ageing. 2021;50(5):1675-1681. doi:10.1093/ageing/afab068 33945608 PMC8437075

[37] Chou MH, Wang JY, Lin CL, Chung WS. DMARD use is associated with a higher risk of dementia in patients with rheumatoid arthritis: a propensity score-matched case-control study. Toxicol Appl Pharmacol. 2017;334:217-222. doi:10.1016/j.taap.2017.09.014 28927738

[38] Newby D, Prieto-Alhambra D, Duarte-Salles T, . Methotrexate and relative risk of dementia amongst patients with rheumatoid arthritis: a multi-national multi-database case-control study. Alzheimers Res Ther. 2020;12(1):38. doi:10.1186/s13195-020-00606-5 32252806 PMC7137292

