# Supplementary material for: Hospital-Diagnosed Infections, Autoimmune Diseases, and Subsequent Dementia Incidence
Source: JAMA Netw Open. 2023 Sep 7;6(9):e2332635. doi: 10.1001/jamanetworkopen.2023.32635 (PMC10485730; doi:10.1001/jamanetworkopen.2023.32635)
Supplement: Supplement 1. — eAppendix. Supplemental Methods eTable 1. ICD Codes for Infections Definition, by Infection Site eTable 2. ICD Codes for Autoimmune Diseases Definition, by Disease Type eTable 3. ICD and ATC Codes for Dementia Definition eTable 4. ICD and ATC Codes for Chosen Comorbidities eFigure 1. Data Analysis Illustration eFigure 2. Population Flow Chart eTable 5. Infection Sites and Subsequent Dementia (Common Reference = No Infection) eTable 6. Autoimmune Disease Types and Subsequent Dementia (Common Reference = No Disease) eFigure 3. Sensitivity Analyses eReferences [file jamanetwopen-e2332635-s001.pdf]

Supplemental Online Content

Janbek J, Laursen TM, Frimodt-Møller N, et al. Hospital-diagnosed infections, autoimmune diseases, and subsequent dementia incidence. *JAMA Netw Open*. 2023;6(9):e2332635. doi:10.1001/jamanetworkopen.2023.32635

eAppendix. Supplemental Methods

eTable 1. *ICD* Codes for Infections Definition, by Infection Site

eTable 2. *ICD* Codes for Autoimmune Diseases Definition, by Disease Type

eTable 3. *ICD* and ATC Codes for Dementia Definition

eTable 4. *ICD* and ATC Codes for Chosen Comorbidities

eFigure 1. Data Analysis Illustration

eFigure 2. Population Flow Chart

eTable 5. Infection Sites and Subsequent Dementia (Common Reference = No Infection)

eTable 6. Autoimmune Disease Types and Subsequent Dementia (Common Reference = No Disease)

eFigure 3. Sensitivity Analyses

eReferences

This supplemental material has been provided by the authors to give readers additional information about their work.

© 2023 Janbek J et al. *JAMA Network Open.*

eAppendix. Supplemental Methods

1. Data management

1.1. ICD codes process and categorization.

For ICD-8 and ICD-10 infection codes, the process was as follows:

We relied on the work we've done in our previous studies[1] with some refinements and additionally including ICD-8 codes, and so the full method is described here.

We scanned through previous studies for ICD-8 and 10 codes using Danish data as well as WHO's official ICD-10 manual and the Danish Health Data Authority's official lists.[2] Expert knowledge in infectious diseases was used to guide the process of including or excluding codes as well as scanning through the Danish webpage sundhed.dk and pubmed publications, as follows:

- Excluded: all codes from the ICD-10 chapters Neoplasms (C); Mental and behavioural disorders (F); Certain conditions originating in the perinatal period (P); Congenital malformations, deformations and chromosomal abnormalities (Q); External causes of morbidity and mortality (V and Y); and Codes for special purposes (U). From ICD-8 chapters excluded were: Neoplasms (140-239), Mental and behavioural disorders (290-315); vitamin and nutritional deficiencies (260-269); Other metabolic diseases (270-279); Hereditary and familial diseases of nervous system (330-333); Other diseases of central nervous system (340-349); Congenital anomalies (740-759); and Delivery (650-662).
- All codes from ICD-10 chapters A and B and ICD-8 chapters 000-136 were included as these indicate infectious disease categories in the ICD manual (135 and 136.02 were exceptions and were not included because these were autoimmune disease codes). All remaining codes were scanned through and excluded were the following: all codes which were not infections and diseases not caused by an infection; all codes where an infection was secondary; an unlikely cause of the disease or where the disease was in majority of reported cases due to non-infectious causes; was due to medication or chemicals (toxic); was due to trauma; was due to allergy/allergic reactions; was due to or related to autoimmune diseases. Granulomas and eosinophilic infections were also excluded.
- Unspecific infections, and diseases due to several causes including an infectious cause were included in our analyses and removed in one sensitivity analysis.

A list of all ICD-10 codes was agreed on by 2 of the authors (JJ, NFM). All codes were then categorized according to infection sites based on the official ICD-10 chapters and guided by expert knowledge, to limit misclassification error for diagnostic codes of specific infections within each site. Categorization was made as follows:

- To categorize all A and B ICD-10 codes, and 000-136 ICD-8 codes: we chose the codes that occurred in our population at least 500 times and categorized those per site categories. The remaining less occurring codes were put together under the category "Less occurring and others".
- Codes for sepsis and herpes were put in separate site categories and taken out from the other site categories. All sepsis and herpes A and B codes and codes from 000-136 were included regardless of their occurrence frequency in our population.
- The category "Less occurring and others" contains the less recurring ICD-10 A and B infection codes in our population; the less occurring codes from the ICD-8 000-136 category (described below); Pregnancy-related infections; and infection codes that did not fit in any site category.

For ICD-8 and ICD-10 autoimmune codes, the process was as follows:

- We relied on previous coding algorithms of autoimmune diseases in studies using the Danish National registry data. We relied on Eaton et al 2010[3] and Harpsøe et al 2014[4] lists of ICD-8 and ICD-10 code of autoimmune diseases, and made refinements where needed depending on our criteria and relying on the expertise of one of our authors. Additionally, we cross checked all codes with a list on the Danish Health Data Authority's website of all ICD-10 codes applied in Denmark, together with any changes made to these codes over time.[2]
- Criteria applied were: inclusion of only chronic and systemic autoimmune diseases, exclusion of codes where an autoimmune disease was due to medication, and exclusion of autoimmune diseases that were mainly in children and young adults. These excluded the following autoimmune diseases (in addition to other sub-category diagnoses in the included diseases): Guillain-Barre syndrome, Alopecia areata, Vitiligo, Juvenile arthritis, Kawasaki syndrome, type 1 Diabetes, Rheumatic fever, Raynaud's phenomenon, and Reiter's disease. Further,

© 2023 Janbek J et al. *JAMA Network Open.*

we did not additionally include autoimmune encephalopathies as they are very rare and are not systemic in a way that would affect the inflammatory processes. Furthermore, the symptoms are cognitive therefore it is difficult to detangle from dementia. Similarly, we did not include neuropathies (e.g. Chronic inflammatory demyelinating polyneuropathy) as these are also not systemic.

- Categories of disease analyzed were made depending on the frequencies in the study population.

1.2. Hospital records management

In the Danish National Patient Register, each person may have several registered hospital records on the same date, where administrative processes or transfers between hospital departments caused these multiple records. We combined records in such cases to result in one hospital contact per person that represents the full hospital stay. Within each infection site and autoimmune disease type, where an individual had a registered start date (in/outpatient) on the same date as a previous discharge date or a previous start date (+1 day), these were considered one hospital contact where we used the first start date and last discharge date to define the period of the contact. Inpatient contacts always triumphed outpatient contacts, in situations where the combined records included both in and outpatient registrations. Resulting dataset was used in analysis models for burden of infections and burden of autoimmune diseases, and all sensitivity analysis models, each further described in the following.

1.3. Infections' burden

After applying the method described in 1.2., we calculated the burden in two ways:

- Number of new infections: To avoid counting several readmissions for the same infection/infectious episode as several diagnosed infections, we chose an arbitrary period of 90 days in between contacts to count separate infections. Within each site category, an infection is defined as each hospital-contact with a start date >90 days following a prior discharge date of another contact with an infection of the same site. Following this process within each infection site category, we will pool the resulting hospital contacts into one dataset which will be used to count the number of infections for each person. If the same hospital contact had 2 diagnoses for infections in two different sites, then that counts as 2. One recurring infectious episode (multiple contacts over a 90-day period) will count as 1. The following figure illustrates this method with an example.

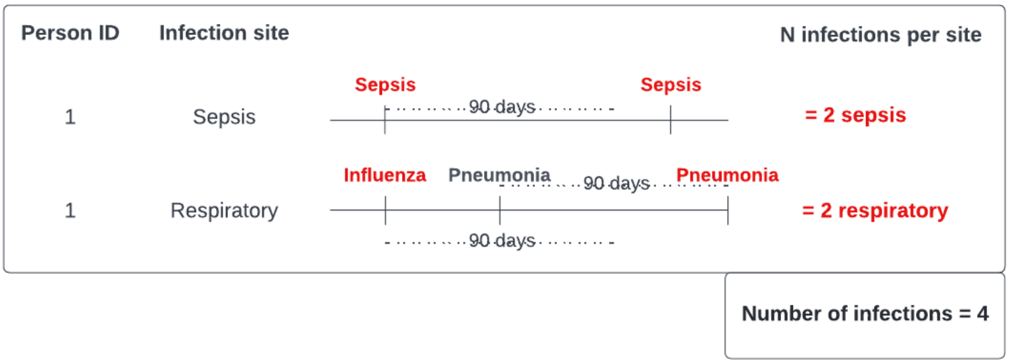

- Number of episodes that led to inpatient admissions: Here we counted each inpatient admission as 1 regardless of the period in between admissions (although admissions cannot be on the same day or +1 day as these were grouped as described in 1.2.). Outpatient contacts with infections were grouped in one category "No inpatient admissions" and represented stand-alone contacts that did not occur on the date (or +1 day) of an inpatient admission. Here, if 2 admissions occurred during the same hospital contact but for 2 different infection sites, then it is counted as 1 admission. This is because 2 recorded admissions for 2 different infections (e.g. sepsis and pneumonia) during the same hospital contact is treated as 1 admission where transfers between departments occurred.

1.4. Autoimmune disease burden

After applying the method described in 1.2., we calculated the burden in two ways:

- Number of different types of autoimmune diseases: Each type of autoimmune disease is counted only once, and a person can only switch from 1 disease to 2 diseases if the second disease was different from the first. If the same hospital contact had 2 diagnoses for 2 different types of autoimmune diseases, then that counts as 2. Repeated hospital contacts for the same disease will result in counting the first only. The following figure illustrates this method with an example.

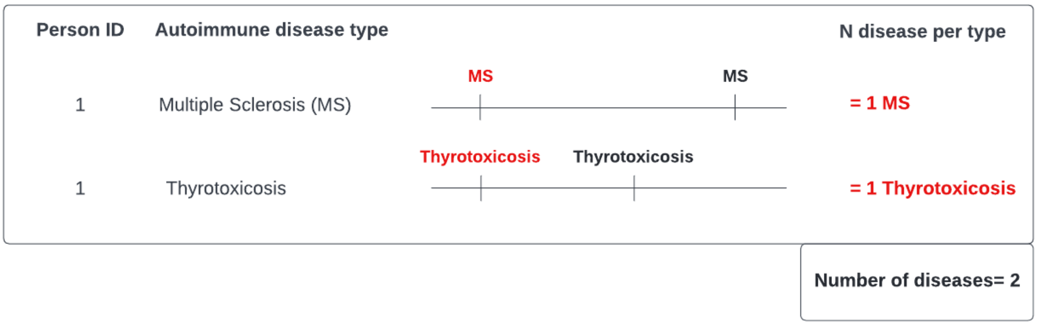

- Number of episodes that led to inpatient admissions: Here we counted each inpatient admission as 1 regardless of the period in between admissions (although admissions cannot be on the same day or +1 day as these were grouped as described in 1.2.). Outpatient contacts with autoimmune disease were grouped in one category "No inpatient admissions" and represented stand-alone contacts that did not occur on the date (or +1 day) of an inpatient admission. Here, if 2 inpatients admissions occurred during the same hospital contact but for 2 different autoimmune disease types, then it is counted as 1 hospital admission.

© 2023 Janbek J et al. *JAMA Network Open.*

eTable 1. ***ICD*** **Codes for Infections Definition, by Infection Site**

Infection site	Code	
	**ICD8**	**ICD10**
Sepsis	038*, 63109, 63110, 63111, 63119, 63129, 63139, 64000, 64002, 64010, 64012, 64020, 64022, 64090, 64092, 64100, 64102, 64110, 64112, 64120, 64122, 64130, 64132, 64140, 64142, 64150, 64152, 64160, 64162, 64170, 64172, 64190, 64192, 64209, 64229, 64239, 64300, 64302, 64380, 64382, 64390, 64392, 64490, 64492, 64500, 64502, 64510, 64512, 64520, 64522, 64530, 64532, 64540, 64542, 64550, 64552, 64560, 64562, 64570, 64572, 64580, 64582, 64590, 64592, 67003, 67008, 67009	R572, J950A, T880A, T802D, T802D1, T814D, O080U, O859, O883, A021, A282B, A327, A392A, A40*, A41*, A427, A548G, B377
Eye and adnexa	36000, 36004, 36005, 36008, 36009, 36108, 36109, 362*, 36300, 36308, 36309, 36390, 36391, 36392, 36393, 36394, 36395, 36398, 36399, 365*, 36600, 36602, 36603, 36604, 36700, 36701, 36702, 36709, 36800, 36801, 36805, 36806, 36808, 36809, 36900, 36901, 36903, 36904, 36905	H000*, H010, H018, H019, H030, H031, H040, H043*, H044, H044A, H050, H050A, H050B, H050C, H050D, H050E, H051, H061, H100, H102, H103, H104, H105, H108, H109, H130, H131, H132, H150, H151, H160A, H161*, H162*, H163, H168, H169, H181, H184B, H192, H192L, H192M, H193*, H220, H300*, H301*, H302, H309, H308, H320, H350E, H440*, H441, H441A, H441E, H451, H469C, H469A, H481, B309
Ear	38000, 38001, 38003, 38008, 38009, 381*, 382*, 383*, 38400, 38401, 38402	H600*, H601*, H602, H603*, H608*, H609, H610*, H620, H621, H622, H623, H624, H65*, H66*, H67*, H680, H70*, H730*, H731, H750, H812, H830, H940
Cardiovascular	420*, 421*, 422*, 42301, 42302, 45702, 45703	I301*, I308, I309, I32*, I33*, I38*, I398, I40*, I410, I411, I412, I430, I514, I514B, I520, I521, I681, I880A, I891*
Respiratory	460*, 461*, 462*, 463*, 464*, 465*, 46600, 46601, 46608, 46609, 470*, 471*, 472*, 473*, 474*, 480*, 481*, 482*, 483*, 484*, 485*, 486*, 49000, 49009, 49101, 49104, 50002, 501*, 50209, 50210, 50211, 50212, 50213, 50215, 50218, 50219, 50300, 50301, 50302, 50303, 50304, 50305, 50306, 50308, 50309, 50699, 50800, 50801, 50802, 50803, 50805, 510*, 513*, 51400, 51990, 01101, 01229	J00*, J01*, J02*, J03*, J04*, J05*, J06*, J09*, J10*, J11*, J12*, J13*, J14*, J15*, J16*, J17*, J18*, J20*, J21*, J22*, J31 *, J32*, J340, J340A, J340B, J340D, J340I, J340J, J350, J36*, J37*, J383C, J383D, J387F, J387G, J390*, J391*, J398A, J40*, J41*, J429A, J429B, J440, J441, J659, J85*, J86*, J985D, J986D, A150, A159, A169, A310, A319, A481, B440, B441
Gastrointestinal	52209, 52219, 52249, 52259, 52269, 52309, 52319, 52339, 52349, 52649, 52720, 52721, 52722, 52728, 52729, 52739, 52800, 52801, 52808, 52809, 52830, 52838, 52839, 52850, 52900, 52901, 52903, 52908, 52909, 52929, 53090, 53091, 53098, 540*, 541*, 542*, 56212, 566*, 567*, 57000, 57001, 57008, 57009, 572*, 57303, 57404, 57405, 57502, 57504, 57508, 57509, 57703, 00919, 00920, 00921, 00999, 02790, 07009	K040*, K041*, K044, K045, K046 , K046A, K047*, K050, K051, K051A, K051B, K051C, K052*, K053*, K102, K102A, K102B, K102C, K102D, K102G, K102H, K112*, K113*, K121*, K122*, K130A, K130B, K130C, K130D, K130E, K130F, K130G, K140, K140A, K140B, K140D, K209, K209A, K221C, K230, K231, K298, K35*, K36*, K37*, K401, K404, K411, K414, K421, K431, K434, K437, K441, K451*, K461, K570*, K572*, K573E, K573F, K574, K578, K61*, K628I, K628L, K628N, K630, K650*, K658, K658A, K658F, K658I, K659, K67*, K720A, K720E, K720F, K750*, K751, K759A, K770, K803*, K804A, K804C, K804E, K810A, K810B, K810C, K810D, K830, K830A, K830B, K830C, K830D, K830E, K830G, K858A, K858D, K858E, K858F, K861A, K930, K931, A020, A045, A047, A049, A081, A084, A090, A099, B159, B169, B178, B181, B182, B199, B370, B378C
Skin	680*, 681*, 682*, 683*, 684*, 68501, 68600, 68608, 68609, 68691, 68695, 68696, 68699, 70402, 03599	L00*, L01*, L02*, L03*, L04*, L050, L08*, L303, L738, L738A, L738H, A469, B079, B079B, B351, B353, B359
Musculoskeletal	710*, 71491, 72000, 72001, 72002, 72003, 72008, 72008, 72010, 72011, 72012, 72013, 72018, 72019, 72029, 72031, 73103, 73299, 04499	M00*, M01*, M462, M463*, M464, M465*, M490, M491, M492, M493, M600*, M601, M608*, M609, M630, M631, M632, M650, M651, M680, M680G, M680H, M710, M711, M726, M729A, M730, M731, M792B, M86*, M900, M901, M902, B919
Urinary	590*, 59320, 59351, 59500, 59501, 59502, 59508, 59509, 59700, 59703, 59708, 59709, 59900, 59901, 59906, 59907	N080, N10*, N11*, N12*, N136*, N151*, N160, N200I, N201I, N209A, N220, N291, N300, N302, N308, N308A, N308B, N308C, N308E, N308F, N308G, N308J, N308K,

© 2023 Janbek J et al. *JAMA Network Open.*

Infection site	Code	
	**ICD8**	**ICD10**
		N309, N330, N340*, N341, N342, N342G, N343, N370, N390, R827, R827B
Genital	601*, 604*, 60739, 60749, 60759, 60773, 61100, 61101, 61108, 61109, 612*, 613*, 614*, 61519, 61600, 61601, 61602, 61603, 620*, 62200, 62201, 62202, 62203, 62208, 62209, 62211, 62212, 62213, 62214, 62215, 62216, 62218, 62219, 62949, 11201, 13100	N41*, N431, N449C, N45*, N481*, N482*, N490, N491, N491A, N491B, N492*, N498*, N499, N511K, N511L, N512, N61*, N70*, N71*, N72*, N730*, N731*, N732*, N733*, N734*, N735*, N738*, N739, N74*, N760, N760B, N760C, N760D, N760E, N761*, N762*, N763*, N764*, N765, N766*, N768, N768A, N768B, N768C, N77*, A630, B373
Nervous	320*, 322*, 323*, 32400, 32408, 32409, 352*, 35399, 355*, 04599	G00*, G01*, G02*, G03*, G04*, G05*, G060, G060A, G060B, G060C, G060E, G060F, G061, G061A, G061C, G061F, G062*, G07*, G08*, G500C, G500D, G518D, G630, G734, G940, A869, A879, B259
Surgical		T793, T801, T802, T802A, T802G, T814, T814A, T814B, T814C, T814E, T814F, T814G, T814H, T814I, T814J, T814P, T814P1, T814U, T814X, T826*, T827*, T835*, T836*, T845*, T846*, T847, T857*, T874, T880
Less occurring and others	Y41*, 28940, 630*, 63481, 635*, 63991, 67000, 67001, 67002, 67004, 67800, 67801, 67803, 67809, 76129, 76139, 76149, 76309, 76319, 76399	Z21*, Z229, D733, D762, E321, E236A, O030, O035, O080, O080B, O080O, O23*, O268D, O353*, O358A, O358B, O411*, O859A, O859B, O860*, O861*, O862*, O863, O863A, O863B, O868, O91*, O980, O981, O982, O983, O984, O985*, O986, O988, O988A, O988C, O988D, O988E, O988F, O988G, O988H, O989, all other A, B, 000-136 codes not categorized elsewhere.
Herpes	052*, 053*, 054*	H191*, O988B, A60*, B00*, B01*, B02*

Note: If the code further includes subcodes and all are also included, then this is denoted by a star (*) after the code. If there are no subcodes or if specific ones are included, then these are listed. For ICD-8 codes the 5-digit code is the most detailed, and no subcodes exist, while for ICD-10 codes the 3-digit code and letter is the most detailed, and no subcodes exist. All codes unspecific to an infection alone: ICD-10: H181, H184B, H193*, H300A, H300B, H301*, H302, H308, H309, H350E, H469C, H481, H830, I308, I309, I328, I408, I409, I514, I514B, J182, J409, J441, J985D, K130C, K130D, K130E, K209, K298, K720A, K720E, K720F, K759A, M608, M609, M792B, N118, N343, N499, N619, G049B, G049C, Z229, R827, R827B. ICD-8: 365*, 36603, 36701, 36702, 38401, 42001, 42008, 42299, 42302, 49009, 51400, 51990, 52850, 53090, 53091, 57000, 57001, 57008, 57009, 57303, 73103, Y41*

© 2023 Janbek J et al. *JAMA Network Open.*

eTable 2. ***ICD*** **Codes for Autoimmune Diseases Definition, by Disease Type**

Categories	Autoimmune disease	ICD8	ICD10	N people
Grouped	Pernicious anemia	2810*	D510	1571
	Autoimmune hemolytic anemia	28390–91	D591	1097
	Idiopathic thrombocytopenic purpura	44649	D693	1908
Grouped	Autoimmune thyroiditis	24503	E063*	3015
	Primary adrenocortical insufficiency (Addison's)	2551*	E271*	1137
One group	Multiple sclerosis	340*	G35*	5652
One group	Iridocyclitis	364*	H20*	7026
Grouped	Crohn's disease	56301	K50*	6656
	Ulcerative colitis	56319	K51*	16 060
	Celiac disease	26900	K900*	2148
Grouped	Autoimmune hepatitis	57193	K73*	1852
	Primary biliary cirrhosis	57190	K743*	1196
Grouped	Pemphigus	69401, 69402, 69403, 69404, 69408, 69409	L100, L101, L102, L103, L104, L108*, L109	401
	Pemphigoid	69405	L12*	1384
	Psoriasis vulgaris	69609–10, 69619	L400*, L401*, L402*, L403*, L408*, L409	12 866
Grouped	Seropositive rheumatoid arthritis	71219, 71239, 71259	M05*, M06*	32 374
	Wegener's granulomatosis	44629	M313	1454
	Dermatopolymyositis	716*	M33*	1078
	Polymyalgia rheumatica	44630–31, 44639	M315*, M316*, M353	26 734
	Systemic sclerosis	7340*	M340, M341, M348*, M349	1521
	Systemic lupus erythematosis	73419	M321, M329	1714
	Sjogren's syndrome	73490	M350*	4300
	Ankylosing spondylitis	71249	M45*	2346
Grouped	Myasthenia gravis	73309	G700	1037
	Behcet's disease	13602	M352	116
	Buerger's syndrome	44319	M311B, I731	226
	Dermatitis Herpetiformis	69309	L130	159
	Erythema nodosum	69529	L52*	665
	Goodpasture's syndrome	44619	M310A	40
	Henoch-Schonlein purpura	28709	D690B	117
	Localized lupus erythematosus	69549	L93*	1342
	Localized scleroderma	70101, 70108, 70109	L940*, L941, L943	560
	Polyarteritis nodosa	44609	M300	663
	Sarcoidosis	135*	D86*	4817
	Sympathetic ophthalmia	36602	H441B	20

Note: If the code further includes subcodes and all are also included, then this is denoted by a star (*) after the code. If there are no subcodes or if specific ones are included, then these are listed. For ICD-8 codes the 5-digit code is the most detailed, and no subcodes exist, while for ICD-10 codes the 3-digit code and letter is the most detailed, and no subcodes exist. Categories were made by combining diseases that were too rare to be assessed separately.

© 2023 Janbek J et al. *JAMA Network Open.*

eTable 3. *ICD* and ATC Codes for Dementia Definition

Dementia type	ICD-8 code	ICD-10 code
Alzheimer's disease	29010	F00*, G30*
Vascular dementia	29309-19	F01*
Frontotemporal dementia	29011	F020, G310A, G310B
Dementia without specification	29009, 29019	F03*, G319
Other dementias	29018	G318, G318E, F021-F028
**Medication**	**ATC code**	
Cholinesterase inhibitors		
Donepezil	N06DA02	
Rivastigmine	N06DA03	
Galantamine	N06DA04	
Glutamate-receptor antagonists		
Memantine	N06DX01	

Note: If the code further includes subcodes and all are also included, then this is denoted by a star (*) after the code. If there are no subcodes or if specific ones are included, then these are listed. For ICD-8 codes the 5-digit code is the most detailed, and no subcodes exist, while for ICD-10 codes the 3-digit code and letter is the most detailed, and no subcodes exist.

eTable 4. *ICD* and ATC Codes for Chosen Comorbidities

Comorbidity	ICD-8 and 10	ATC code
Hypertension	400*, 401*, 402*, 403*, 404*, 41009, 41109, 41209, 41309, 41409, 43509, 43700, 43701, 43708, 43709, 43809, I10*-I13*, I15*	C02*-C04*, C07*-C09*
Diabetes	249*, 250*, E10*-E14*	A10A*, A10B*
Stroke	430*, 431*, 432*, 433*, 434*, 436*, I60*-I64*, I69*	-
Myocardial infarction	41099, I21*, I22*, I23*	-
Hypercholesteremia	27900, E780*	C10*

For prescriptions, the comorbidity was defined if a person redeemed a minimum of two prescriptions of the ATC codes, at any time during the study period. This was done to ensure the comorbidity was present, in the absence of diagnostic codes in the primary care setting. The date of the comorbidity is the first diagnosis or prescription.

© 2023 Janbek J et al. *JAMA Network Open.*

eFigure 1. Data Analysis Illustration.

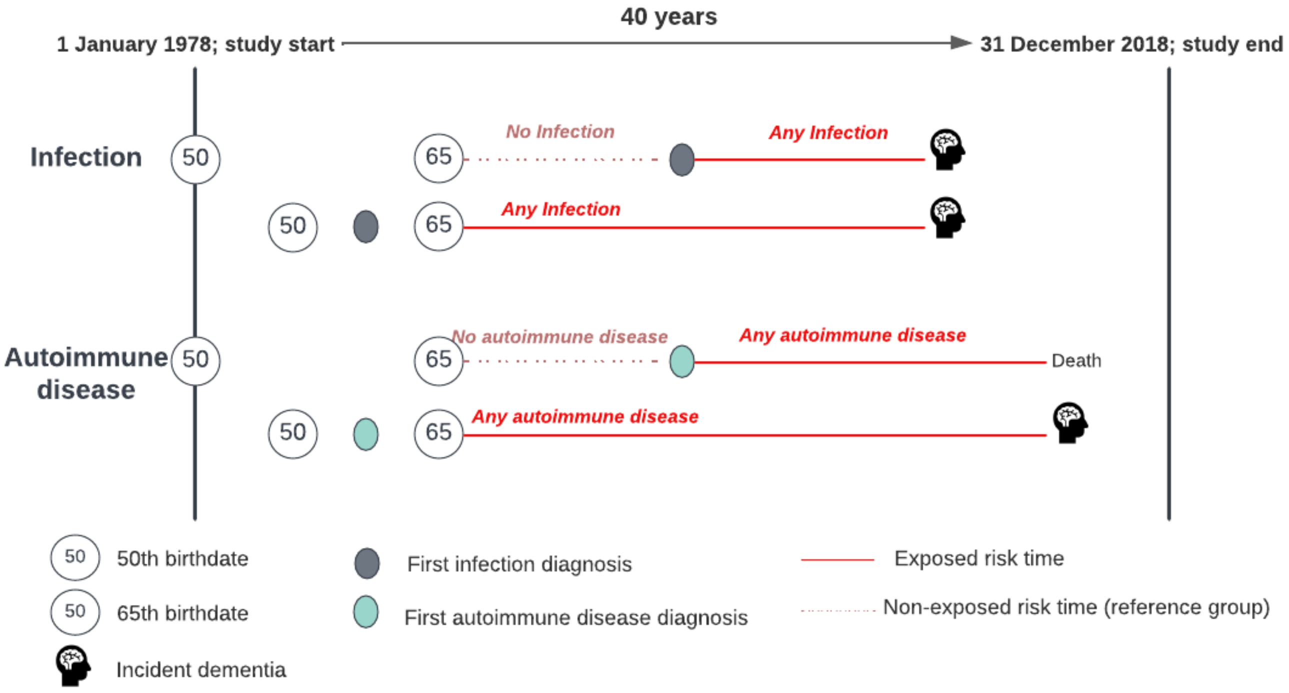

© 2023 Janbek J et al. *JAMA Network Open.*

eFigure 2. Population Flow Chart.

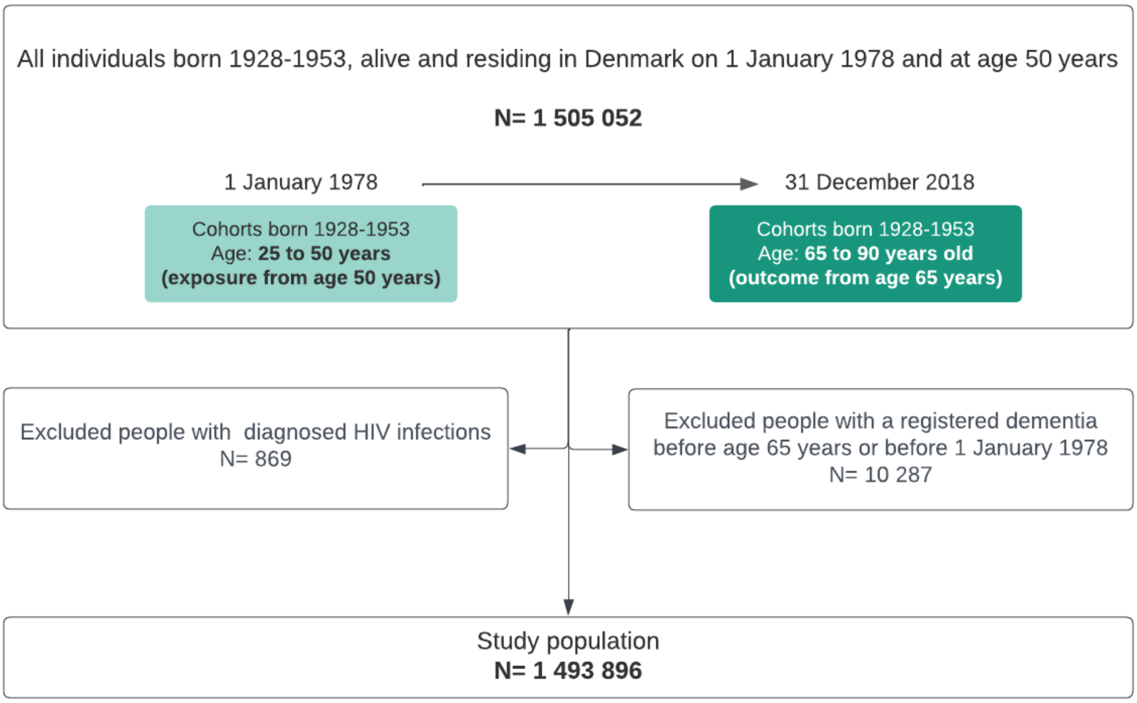

The birth cohorts were chosen because younger cohorts born before 1928 would not have exposure information from age 50 years and older cohorts born after 1953 would not have the chance to be at risk for the outcome by the end of the study period (age at risk was defined as ≥65 years).

© 2023 Janbek J et al. *JAMA Network Open.*

eTable 5. Infection Sites and Subsequent Dementia (Common Reference = No Infection)

Infection site	Pyrs at risk	N dementia	Rate/ 1000 pyrs	Fully adjusted IRR (95% CI)
Urinary	814 528	12 542	**15.4**	**2.04 (2.00- 2.08)**
Other infections	3 424 304	23 982	7.0	1.33 (1.31- 1.35)
Sepsis	238 147	3558	**14.9**	**1.98 (1.91- 2.05)**
Other infections	4 000 685	32 966	8.2	1.46 (1.44- 1.48)
Nervous	55 748	631	**11.3**	**1.80 (1.67- 1.95)**
Other infections	4 183 084	35 893	8.6	1.49 (1.47- 1.51)
Respiratory	1 579 158	16 076	**10.2**	**1.61 (1.58- 1.64)**
Other infections	2 659 674	20 448	7.7	1.42 (1.39- 1.44)
Others, less occurring	380 915	3998	**10.5**	**1.66 (1.61- 1.72)**
Other infections	3 857 917	32 526	8.4	1.48 (1.45- 1.50)
Skin	692 463	5841	**8.4**	**1.52 (1.48- 1.56)**
Other infections	3 546 369	30 683	8.7	1.49 (1.47- 1.51)
Herpes	72 452	673	**9.3**	**1.44 (1.34- 1.56)**
Other infections	4 166 380	35 851	8.6	1.49 (1.47- 1.52)
Gastrointestinal	1 101 213	9166	**8.3**	**1.41 (1.38- 1.44)**
Other infections	3 137 618	27 358	8.7	1.52 (1.50- 1.55)
Musculoskeletal	119 414	968	**8.1**	**1.38 (1.30- 1.48)**
Other infections	4 119 418	35 556	8.6	1.50 (1.47- 1.52)
Eye	267 882	1885	**7.0**	**1.33 (1.27- 1.39)**
Other infections	3 970 950	34 639	8.7	1.50 (1.48- 1.53)
Surgical	214 583	1745	**8.1**	**1.35 (1.28- 1.41)**
Other infections	4 024 249	34 779	8.6	1.50 (1.48- 1.52)
Ear	231 624	1723	**7.4**	**1.33 (1.26- 1.39)**
Other infections	4 007 208	34 801	8.7	1.50 (1.48- 1.53)
Genital	273 905	1942	**7.1**	**1.31 (1.25- 1.37)**
Other infections	3 964 927	34 582	8.7	1.51 (1.48- 1.53)
Cardiovascular	65 440	533	**8.1**	**1.31 (1.20- 1.42)**
Other infections	4 173 392	35 991	8.6	1.50 (1.47- 1.52)
No infection (ref)	9 854 471	39 019	4.0	1

IRR= Incidence Rate Ratio; pyrs= person years at risk; ref= reference group.

© 2023 Janbek J et al. *JAMA Network Open.*

eTable 6. Autoimmune Disease Types and Subsequent Dementia (Common Reference = No Disease)

Disease type	Pyrs at risk	N dementia	Rate/ 1000 pyrs	Fully adjusted IRR (95% CI)
Pemphigus, Pemphigoid, Psoriasis	94 028	762	**8.1**	**1.18 (1.09- 1.27)**
Other autoimmune diseases	796 427	5967	7.5	1.02 (0.99- 1.05)
Anemia, thrombocytopenic purpura	28 263	273	**9.7**	**1.14 (1.00- 1.30)**
Other autoimmune diseases	862 191	6456	7.5	1.03 (1.01- 1.06)
Multiple sclerosis	38 598	238	**6.2**	**1.13 (0.98- 1.30)**
Other autoimmune diseases	851 856	6491	7.6	1.03 (1.01- 1.06)
Crohn's, colitis, Celiac	159 288	1207	**7.6**	**1.11 (1.05- 1.18)**
Other autoimmune diseases	731 167	5522	7.6	1.02 (0.99- 1.05)
Thyroiditis, Addison’s	26 293	179	**6.8**	**1.03 (0.88- 1.20)**
Other autoimmune diseases	864 161	6550	7.6	1.04 (1.01- 1.07)
Hepatitis, biliary cirrhosis	18 839	134	**7.1**	**1.00 (0.83- 1.20)**
Other autoimmune diseases	871 615	6595	7.6	1.04 (1.01- 1.07)
Systemic autoimmune diseases	467 761	3676	**7.9**	**1.00 (0.97- 1.04)**
Other autoimmune diseases	422 693	3053	7.2	1.08 (1.04- 1.12)
Others,less occurring	65 580	475	**7.2**	**1.07 (0.97- 1.17)**
Other autoimmune diseases	824 874	6254	7.6	1.03 (1.01- 1.06)
Iridocyclitis	48 357	296	**6.1**	**0.87 (0.77- 0.98)**
Other autoimmune diseases	842 097	6433	7.6	1.05 (1.02- 1.08)
No disease (ref)	13 202 848	68 814	5.2	1

IRR= Incidence Rate Ratio; pyrs= person years at risk; ref= reference group.

© 2023 Janbek J et al. *JAMA Network Open.*

eFigure 3. Sensitivity Analyses

		N Exposed	N Outcome	Fully adjusted IRR (95% CI)
Calendar year strata				
Any infection	Year <2006		3878	1.61 (1.55- 1.68)
	Year >=2006		32 646	1.48 (1.45- 1.50)
Any autoimmune disease	Year <2006		653	1.06 (0.98- 1.15)
	Year >=2006		6076	1.04 (1.01- 1.07)
Mortality				
Any infection		677 147	38 743	3.63 (3.61- 3.66)
Any autoimmune disease		127 721	7026	1.24 (1.23- 1.25)
With exposures before age 50 years				
Any infection		774 095	239 516	1.45 (1.43- 1.47)
Any autoimmune disease		138 334	37 958	1.04 (1.01- 1.06)
Without codes unspecific to an infection				
Any infection		654 145	35 218	1.49 (1.47- 1.52)

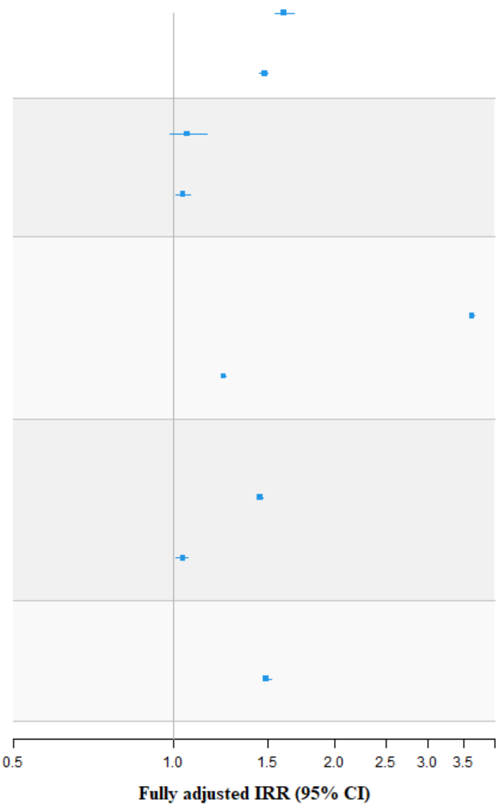

Calendar year strata: Dementia IRRs in two calendar periods to test any risk variation in the early vs late period of our study. Mortality: Mortality rate ratios following any infection (ref= no infection) and any autoimmune disease (ref= no autoimmune disease). With exposures before age 50 years: Dementia IRRs for any infection including infections before age 50 years (ref= no infection) and for any autoimmune diseases including registered autoimmune diseases before age 50 years (ref= no autoimmune disease). Without codes unspecific to an infection: Dementia IRRs for any infection (ref= no infection). Fully adjusted IRRs were adjusted for age, sex, calendar year, highest attained educational level, hypertension, diabetes, hypercholesteremia, myocardial infarction, and stroke, and further adjusted for infections when autoimmune diseases were assessed and vice versa. Error bars represent the 95% CIs. IRRs are presented on a log scale.

eReferences

1. Janbek J, Frimodt-Møller N, Laursen TM, Waldemar G. Dementia identified as a risk factor for infection-related hospital contacts in a national, population-based and longitudinal matched-cohort study. *Nat Aging*. 2021;1(2):226-233. doi:10.1038/s43587-020-00024-0
2. The Danish Health Data Authority. Revision af Klassifikation af sygdomme. Revision af Klassifikation af sygdomme. https://sundhedsdatastyrelsen.dk/da/rammer-og-retningslinjer/om-klassifikationer/sks-klassifikationer/klassifikation-sygdomme/kodeaendringer. Published 2021. Accessed August 23, 2022.
3. Eaton WW, Pedersen MG, Atladóttir HÓ, Gregory PE, Rose NR, Mortensen PB. The prevalence of 30 ICD-10 autoimmune diseases in Denmark. *Immunol Res*. 2010;47(1-3):228-231. doi:10.1007/s12026-009-8153-2
4. Harpsøe MC, Basit S, Andersson M, et al. Body mass index and risk of autoimmune diseases: a study within the Danish National Birth Cohort. *Int J Epidemiol*. 2014;43(3):843-855. doi:10.1093/ije/dyu045

© 2023 Janbek J et al. *JAMA Network Open.*
