# Supplementary material for: Hospital-Diagnosed Infections, Autoimmune Diseases, and Subsequent Dementia Incidence
Source: JAMA Netw Open. 2023 Sep 7;6(9):e2332635. doi: 10.1001/jamanetworkopen.2023.32635 (PMC10485730; doi:10.1001/jamanetworkopen.2023.32635)
Supplement: Supplement 2. — Data Sharing Statement [file jamanetwopen-e2332635-s002.pdf]

Data Sharing Statement

Janbek. Hospital-Diagnosed Infections, Autoimmune Diseases, and Subsequent Dementia Incidence. *JAMA Netw Open*. Published September 07, 2023.
doi:10.1001/jamanetworkopen.2023.32635

Data
Data available: No

Additional Information
Explanation for why data not available: All data in the study are based on the public Danish health registers, and access to such data is regulated by a set of rules.
